# Gene expression profiling after exposure to a chemical carcinogen, Pentabrominated Diphenyl Ether, at different life stages

**DOI:** 10.3389/ftox.2022.1028309

**Published:** 2023-01-04

**Authors:** Keith R. Shockley, June K. Dunnick

**Affiliations:** ^1^ Biostatistics and Computational Biology Branch, Division of Intramural Research, National Institute of Environmental Health Sciences, Research Triangle Park, Durham, NC, United States; ^2^ Systems Toxicology Branch, Division of Translational Toxicology, National Institute of Environmental Health Sciences, Research Triangle Park, Durham, NC, United States

**Keywords:** pentabrominated diphenyl ether (PBDE), liver transcripts, PND 4, PND 22, adult life stage changes

## Abstract

Exposure to environmental hazards occurs at different stages of our lifetime–infant, child, adult. This study integrates recently published toxicogenomics data to examine how exposure to a known rat chemical carcinogen (pentabrominated diphenyl ether (PBDE)) upregulated liver transcriptomic changes at different life cycle stages (PND 4, PND 22, adult). We found that at all three life cycle stages PBDE exposure induced hepatocellular transcriptomic changes in disease pathways including cancer, metabolic, membrane function, and Nrf2 antioxidant pathways, pathways all characteristics of chemical carcinogens. In addition, in the adult rat after a 5-day exposure to the chemical carcinogen, there was upregulation of members of the Ras oncogenic pathway, a specific pathway found to be activated in the PBDE-induced tumors in rats in a previous hazard identification cancer study. The findings of liver transcript changes characteristic of carcinogenic activity after early life exposures and after short-term adult exposures provides data to support the use of transcriptomic data to predict the apical cancer endpoints in model studies. Using data from gene expression profiling studies after neonatal, young, or adult short-term chemical exposure helps to meet the 21st century toxicology goal of developing study designs to reduce, refine, and replace the use of traditional 2-year rodent cancer studies to provide hazard identification information. The studies reported here find that key transcripts associated with carcinogenesis were elevated in neonate (PND 4), young (PND 22) and adult animals after short-term exposure to PBDE, a known experimental chemical carcinogen in model systems.

## 1 Introduction

In the 21st century, the toxicology community seeks to develop hazard identification information using “modern tools”, including data from gene expression profiling studies (toxicogenomic (TGMX) studies). The U.S. National Academy of Sciences discusses this strategy in the 2007 report Toxicity Testing in the 21st Century: A Vision and a Strategy (U.S. National Research Councilo.t.N.A, 2007). This paper evaluates liver toxicogenomic changes after exposure to a model chemical carcinogen (pentabrominated diphenyl ether (Dunnick et al., 2018a; Dunnick et al., 2018b)) in male rats at PND 4 (after *in utero* exposure), PND 22 (after *in utero*/postnatal exposure), and in adult male rats (after 5-day exposures). The hypothesis for this study was that exposure to pentabrominated diphenyl ether at different life cycle stages would activate disease pathways that could be used to predict longer term toxic and carcinogenic effects, thereby reducing the number of animals needed to obtain toxic and cancer hazard identification information used in risk assessment evaluations (Dunnick et al., 2018c; Shockley et al., 2020a; Dunnick et al., 2020).

For the model carcinogen (pentabrominated diphenyl ether (PBDE)) used in this study, world-wide exposure continues to be of concern because PBDE containing products remain in the home and in the environment (Maddela et al., 2020; Simonetti et al., 2020; Jin et al., 2021), and PBDE exposures may occur during e-waste recycling (Wei et al., 2020; Zhou et al., 2020; Wannomai et al., 2021). As fat-soluble organic pollutants, PBDEs persist in human and animal tissues with long tissue half-lives (Möller et al., 2011; Abbasi et al., 2019; Maddela et al., 2020). The CDC NHANES program (National Health and Nutrition Examination Survey) has collected data on occurrence of these chemicals in human tissues, and exposures to these chemicals continue to be widespread in the United States (Sjödin et al., 2019; Vuong et al., 2020). In humans, exposure to these chemicals has been associated with various health hazards including alteration of thyroid function and developmental changes (Chen et al., 2014; Makey et al., 2016; Sjödin et al., 2020).

Three recent toxicogenomics studies evaluated the gene expression response to PBDE exposure in male rats at PND 4, PND 22, and adult animal evaluations (Dunnick et al., 2018c; Shockley et al., 2020a; Dunnick et al., 2020). Each of these studies had a separate research interest (i.e., the response of male rats to PBDE at either PND4, PND22, or adult). Accordingly, each investigation produced a distinctive dataset derived from the unique study design underlying its own particular scientific focus. These previous analyses were conducted independently and served to expand the field of PBDE research by characterizing the toxicological response to the carcinogenic agent at a specific point in male rat development. This study analyzes and compares the PBDE-induced liver transcriptomic changes at PND 4, PND 22, or in the adult to explore the gene expression response to PBDE in the male rat at different life cycle stages. The new information resulting from this analysis can be used in risk assessment and may help to lay a framework in predicting long-term toxic effects following exposure to environmental agents at different life cycle stages (Harrill et al., 2019).

## 2 Materials and methods

### 2.1 Chemicals

As described in previously reported studies (Dunnick et al., 2018c; Shockley et al., 2020a; Dunnick et al., 2020), the chemical pentabromodiphenyl ether-47 (PBDE-47, 2,2′,4,4′-tetrabromodiphenyl ether; CAS# 5436-43-1) was obtained from Cerillant Corp (Round Rock, TX; Lot ER081208-02). Chemical identity was confirmed by mass spectrometry and nuclear magnetic resonance spectroscopy. The purity for PBDE-47 was 99.6% by gas chromatography (GC) analysis using flame ionization detection. There were no quantifiable pentabrominated dibenzodioxins or furans present in the PBDE-47 sample. PBDE-mixture (DE-71, technical pentabromodiphenyl; CAS# 32534-81-9) was obtained from Great Lakes Corporation (West Lafayette, IN; Lot 2550OA30A). The identity and purity were determined as described previously. The DE-71 composition was: PBDE-99 (41.7%), PBDE-47 (35.7%), PBDE-100 (10.4%), PBDE-154 (3.6%), PBDE-153 (3.3%), and PBDE-85 (2%); low levels of pentabrominated dibenzodioxins and furans were also identified (approximately 7 × 10^–6^% by weight).

### 2.2 Animals and exposures

The animals used were either male Wistar Han rat or Harlan Sprague Dawley rats which induced transcripts encoding metabolic enzymes, members of the Nrf2 antioxidant pathway, and ABC transporters upon exposure to PBDE-47 and the PBDE-47 mixture as described in previous publications (Dunnick et al., 2018c; Shockley et al., 2020a; Shockley et al., 2020b; Dunnick et al., 2020). In this paper, the transcriptomic data from different life stages was analyzed by groups (Group 1–5, see Table 1). Group One examined rat liver toxicogenomic changes after PBDE-47 exposure for 5 days. In this study adult Harlan Sprague Dawley male rats were exposed to the PBDE mixture by oral gavage in corn oil for five consecutive days; on the sixth day liver samples were taken for toxicogenomic analysis (Shockley et al., 2020a; Shockley et al., 2020b). Group Two and Group Four examined rat liver toxicogenomic changes in PND 4 pups after exposure of dams to PBDE-47 or the PBDE mixture (DE-71), respectively (Dunnick et al., 2020). In these studies, Wistar Han rat dams were dosed with a dosing volume of 5 ml/kg/day from GD 6 through PND 4, 7 days per week. Controls received corn oil vehicles. Liver samples were taken for toxicogenomic analysis in male pups at PND 4. Group Three and Group Five examined liver toxicogenomic changes after exposure of dams and pups to PBDE-47 or the PBDE mixture, respectively (Dunnick et al., 2018c). In these studies, Wistar Han rat dams were dosed with a dosing volume of 5 ml/kg/day from GD 6 through PND 21, 7 days per week. Controls received corn oil vehicles. Pups received daily the PBDE mixture dosing from PND12-21. At PND 22 liver samples from pups were taken for toxicogenomic analysis.

**TABLE 1:** Summary of thyroid hormone levels and liver pathology findings after exposure to PBDE 47 and/or PBDE mixture (DE-71) at different life cycle stages.

	Group 1 (Shockley et al., 2020a)						Group 2 (Dunnick et al., 2020)				Group 3 (Dunnick et al., 2018c)				Group 4 (Dunnick et al., 2020)				Group 5 (Dunnick et al., 2018c)			
Chemical exposure	PBDE 47						PBDE 47				PBDE 47				PBDE mixture (DE-71)				PBDE mixture (DE-71)			
Dosing Schedule	5 days of consecutive dosing in seven-week-old Harlan Sprague Dawley Rats						Pups from Wistar Han rat dams exposed from GD 6 through PND 4 (no direct pup dosing)				Pups from Wistar Han rat dams exposed GD 6 through PND 21; pups received daily dosing from PND 12–21				Pups from Wistar Han rat dams exposed from GD 6 through PND 4 (no direct pup dosing)				Pups from Wistar Han rat dams exposed GD 6 through PND 21; pups received daily dosing from PND 12–21			
TGMX time point	After 5 Day Exposure						PND 4				PND 22				PND 4				PND 22			
Oral gavage (μmol/kg)	0	0.1	1.0	10	100	1000	0	0.2	31	103	0	0.2	31	103	0	0.2	27	89	0	0.2	27	89
Total Thyroxine TT4 (Ig/dL)	5.77 ± 0.63	5.77 ± 0.35	5.00 ± 0.27	5.94 ± 0.28	1.48 ± 0.10**	0.80 ± 0.08**	1.14 ± 0.16	1.18 ± 0.13	0**	0**	5.08 ± 0.21	5.05 ± 0.26	3.20 ± 0.08**	3.00 ± 0.10**	0.96 ± 0.15	1.5 ± 0.15	0.59 ± 0.11	0**	5.67 ± 0.36	5.67 ± 0.31	3.66 ± 0.19**	2.69 ± 0.23**
T4 (% control)	-	100	86	102	25	14	-	104	0	0	-	99	63	59	-	157	61	0	-	100	65	48
Treatment related liver lesions					+	++							+	+			+	+		+	+	+

** *p* = < 0.01 (treated group vs. control group).

Pathology Designation: + (minimal hepatocyte hypertrophy and/or central vacuolization); ++ (moderate or marked hepatocyte hypertrophy and/or central vacuolization).

### 2.3 Microarray, RNA extraction method, and statistical analysis

The methods for liver transcriptomics were similar across the different life cycle exposure scenarios**.** In all cases the left lateral lobe of the liver was processed for RNA isolation. Liver RNA was isolated from the liver tissue, amplified and biotin labeled, and hybridized to Affymetrix Rat Genome 230 2.0 arrays (Affymetrix, Santa Clara, CA) as described previously (Dunnick et al., 2018c; Shockley et al., 2020a; Dunnick et al., 2020). Each GeneChip^®^ array was scanned using an Affymetrix GeneChip^®^ Scanner 3000 7G to generate raw expression level data (.CEL files). Probe intensity data from all Rat Genome 230 version two Affymetrix GeneChip^®^ arrays were read into the R software environment (http://www.R-project.org) directly from. CEL files using the R/affy package (Gautier et al., 2004). Normalization was carried out using the robust multi-array average (RMA) method separately for each Group.

Statistical contrasts were used to find pairwise gene expression differences between the control group and each dose group using the R/maanova package (Wu et al., 2003). For each flame retardant, the model

$$Y_{i}=\mu +DOSE+\varepsilon_{i}$$ (1)

was used to fit the log_2_ transformed gene expression measures Y_i_, where *µ* is the mean for each array and *ε_i_* captures random error for probe set *i*. All statistical tests were performed using F_s_, a modified F-statistic incorporating shrinkage estimates of variance components (Cui et al., 2005). To reduce the number of false positives, *p*-values were adjusted for multiple hypothesis testing corresponding to all probe sets on the array using the Benjamin-Hochberg false discovery rate (FDR) procedure implemented using the p.adjust() function in R. This correction controls the expected proportion of errors among the significant results (Benjamini and Hochberg, 1995). Unless otherwise noted, an FDR threshold of 0.05 was used for statistical significance. Log_2_ fold changes for each exposure group were calculated by subtracting the control (0 μmol/kg) and dose treated relative expression values obtained from model (1) above (Churchill, 2004).

Overrepresented canonical pathways were determined from each gene list obtained above by testing for association with gene pathway relationships (www.ingenuity.com). Pathway enrichment was determined based on the one-tailed Fisher exact test, where *p*-values were adjusted for multiple testing using the FDR approach described above.

The transcriptomic data for these studies can be found on the National Library of Medicine’s GEO database web site. The Affymetrix data for each dataset can be accessed from GEO using GSE153366 for Group One, GSE154914 for Group Two and Group Four, and GSE124431 for Group Three and Group Five.

The benchmark dose (BMD) is defined as the dose corresponding to a stimulated change in response referred to as the benchmark response (BMR). Liver transcriptomic data were used to calculate the BMD and the lower bound of the 95% confidence interval of the BMD using BMDExpress version 2.0 (Phillips et al., 2019). All BMD calculations were performed within the BMDExpress framework separately for each combination of chemical and Group.

The BMD analysis followed the criteria described in the Genomic Dose Response recommendations (National Toxicology Program, 2018). First, we determined whether there was adequate signal in each dataset. Control-AFFX probe sets were first removed from each dataset, and a classical one-way ANOVA was used to filter the remaining RMA-normalized probe set intensities to find transcripts that were differentially expressed across experimental groups with a *p*-value <0.05. The presence of differentially expressed transcripts indicated that the dataset contained adequate signal. Next, for datasets with adequate signal, we used a bootstrap version of Williams’ trend test to find probe sets with dose group changes relative to the control group using a *p*-value <0.05 and a fold change of at least 1.5 computed with 10,000 permutations of group labels. Next, the Hill, power, linear, second-degree polynomial, and a set of four exponential models were fit to the data for each remaining probe set. The BMR level was set to 1 standard deviation above or below the control group. The lowest Akaike information criterion (AIC) was used to select the best fitting model. Hill model fits were not selected if the estimated dose at half maximal response was less than 1/3 of the lowest positive dose, and the next best model was selected instead.

The calculated BMD values are used as input data for Gene Ontology (GO) analyses. When more than one probe set mapped to the same Entrez ID, the BMD values were averaged across probe sets to obtain a single expression value for each Entrez ID. Probe sets that mapped to more than one Entrez ID were removed from the analysis. The resulting Entrez IDs were matched to Biological Process GO terms as a basis for gene set definitions. The output consists of a range of summary exposure levels (mmol/kg/day) for BMD and BMDL for each category computed from the BMD and BMDL values for the genes in a category. Probes were removed if BMDU/BMDL >40 with a fit *p*-value <0.1 and if BMD > highest dose level. The final GO Biological Process results were further processed to have ≥3 genes and ≥5% of the genes in the category with genes that passed the filtering scheme described above.

## 3 Results

Exposure of the male rat to PBDE-47 or PBDE mixture at different life cycle stages (PND 4, PND 22, and adult) caused treatment-related rat liver transcript changes (Table 2). Generally, there were more transcripts upregulated than downregulated for each of the exposure groups. At approximately 100 mmol/kg treatment, the number of significant transcripts was greatest in the adult animal, followed by PND 22 animals, and finally in PND 4 animals. While this study uses a false discovery rate significance threshold of 0.05, the trends described above were consistent across a wide range of significance thresholds (Table 2). In each age group, the number of significant transcripts increased with increasing exposure to PBDE. At PND 4 and PND 22, there were more significant transcripts after PBDE 47 exposure than after exposure to the PBDE mixture at the same exposure levels, except at PND 4 at 100 mmol/kg treatment in which there was a comparable number of changes.

**TABLE 2:** Number of significant transcripts induced in the liver by pentabrominated diphenyl ether (PBDE) or PBDE mixture (DE-71).

					False discovery rate (FDR)				
Group	Animals	Chemical	TGMX time point	Dose (μmol/kg)	0.2	0.05	0.01	0.001	10^–10^
1	HSD Rats	PBDE 47	After 5 Days	0.1	280	32	5	0	0
1	HSD Rats	PBDE 47	After 5 Days	1.0	247	22	3	0	0
1	HSD Rats	PBDE 47	After 5 Days	10	479	99	24	7	2
1	HSD Rats	PBDE 47	After 5 Days	100	2162	948	446	195	33
1	HSD Rats	PBDE 47	After 5 Days	1000	4670	2937	2016	1268	496
2	WH pups	PBDE 47	PND4	0.2	139	2	0	0	0
2	WH pups	PBDE 47	PND4	31	342	40	11	3	2
2	WH pups	PBDE 47	PND4	103	655	140	50	20	3
3	WH pups	PBDE mixture	PND4	0.2	2	0	0	0	0
3	WH pups	PBDE mixture	PND4	31	103	13	1	0	0
3	WH pups	PBDE mixture	PND4	103	709	161	24	0	0
4	WH pups	PBDE 47	PND22	0.2	255	13	1	0	0
4	WH pups	PBDE 47	PND22	27	999	263	92	32	13
4	WH pups	PBDE 47	PND22	89	1675	590	240	82	16
5	WH pups	PBDE mixture	PND22	0.2	33	1	0	0	0
5	WH pups	PBDE mixture	PND22	27	318	101	43	6	3
5	WH pups	PBDE mixture	PND22	89	832	232	91	10	5

**FIGURE 1:**
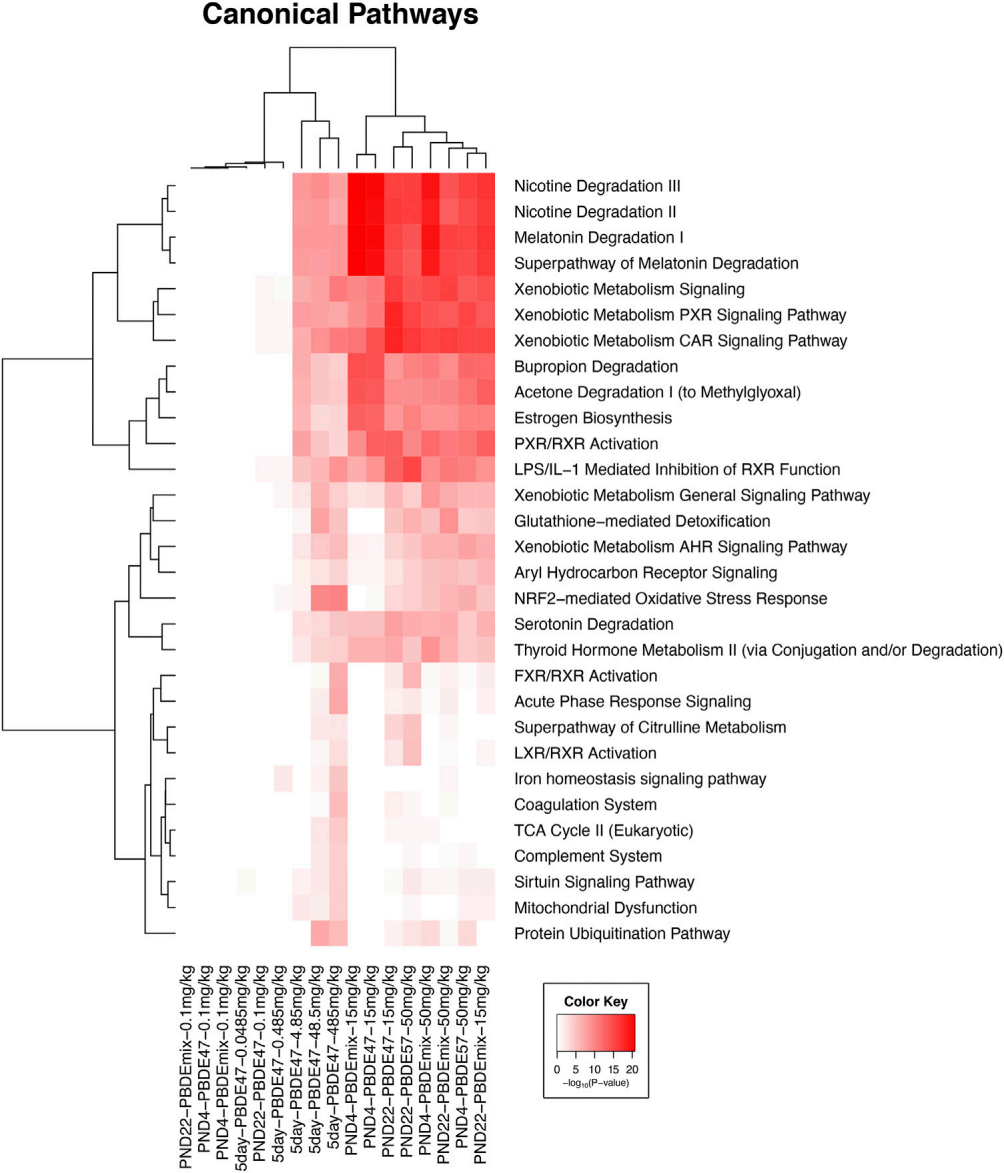
Clustered heat map of log_10_ *p*-values associated with enriched Ingenuity Canonical Pathways associated with the changed transcripts from each experiment in all five groups in this study. Included pathways have at least one significant pathway after multiple test correction (FDR <0.01). The color key scale indicates the minus log_10_ *p*-value of pathway significance.

The significant liver transcripts at all life cycle stages included upregulation of transcripts for liver metabolic phase one and phase two enzymes, NrF2 antioxidant pathway transcripts, and membrane transport protein transcripts (Table 3 and Figure 1). Some of the transcripts with the greatest fold change (compared to controls) were changes in metabolic function transcripts including upregulation of cytochrome activity transcripts for *Cyp2b6, Cyp1a1*, *Cyp3a5*, *Por* (Table 3). The upregulation of UGT transcripts (Table 3) was accompanied by lower levels of thyroid hormone level (T4) after PBDE exposure (Table 1). T4 levels were decreased with increasing PBDE exposure levels in rats at PND4, PND22, and in adult animals, and there were treatment-related liver lesions at the higher exposure levels in rats at all three life cycle stages (Table 1). The liver lesions were most severe in the PBDE adult exposed rats (Shockley et al., 2020a).

**TABLE 3:** Selected PBDE-induced liver transcript alterations associated with metabolic, membrane, NRF2 pathway, and lipid function and cancer pathways.

Gene	Group 1 (Shockley et al., 2020a)					Group 2 (Dunnick et al., 2020)			Group 3 (Dunnick et al., 2018c)			Group 4 (Dunnick et al., 2020)			Group 5 (Dunnick et al., 2018c)		
Chemical Exposure	PBDE 47					PBDE 47			PBDE 47			PBDE mixture (DE-71)			PBDE mixture (DE-71)		
Dosing Schedule	5 days of consecutive dosing in seven-week-old Harlan Sprague Dawley Rats					Pups from Wistar Han rat dams exposed from GD 6 through PND 4 (no direct pup dosing)			Pups from Wistar Han rat dams exposed GD 6 through PND 21; pups received daily dosing from PND 12–21			Pups from Wistar Han rat dams exposed from GD 6 through PND 4 (no direct pup dosing)			Pups from Wistar Han rat dams exposed GD 6 through PND 21; pups received daily dosing from PND 12–21		
TGMX Time Point	After 5-day exposure					PND4			PND22			PND4			PND22		
Oral Gavage (μmol/kg)	0.1	1	10	100	1000	0.2	31	103	0.2	31	103	0.2	27	89	0.2	27	89
																	
*Liver Metabolism*																	
1370269_at *Cyp1a1* cytochrome P450 family 1 subfamily A member 1				6.11***	65.54***		5.66**	10.98***			4.28*		59.68*	91.3*	5.13	60.47***	77.64***
1387243_at *Cyp1a2* cytochrome P450 family 1 subfamily A member 2					1.23*		2.26	2.09		−1.27			13.88	53.83*		2*	2.49*
1369136_at *Cyp2a6* (includes others) cytochrome P450 family 2 subfamily A member 6							5.33*	7.17**		14.54***	23.38***		6.99*	16.93*		14.92*	14.31*
1371076_at *Cyp2Bb* cytochrome P450 family 2 subfamily B member 6			4.51***	10.94***	12.51***		3.98***	4.4***		4.25***	4.52***		3.41*	4.03*		4.89**	4.7**
1370241_at *Cyp2c8* cytochrome P450 family 2 subfamily C member 8					1.28*		5.73*	9.26**		24.91***	31.68***		5.53*	11.15*		46.27**	55.8**
1370580_a_at *Cyp2c19* cytochrome P450 family 2 subfamily C member 19			1.2**	1.34***	1.41***		3.37***	3.49***		1.64***	1.69***		4.34*	4.89*		1.69*	1.72*
1387118_at *Cyp3a5* cytochrome P450 family 3 subfamily A member 5			1.37*	2.21***	2.84***		1.62*	1.71*	−1.23	1.68**	1.84**		1.6*	1.78*		1.96***	1.97***
1387109_at *Por* cytochrome p450 oxidoreductase				2.24*	3.85***		1.48	1.76*		1.76*	1.92*			2.49*		2.1*	2.52*
1368607_at *Cyp4a22* cytochrome P450 family 4 subfamily A member 22				3.32**	4.36***					2.82*	2.57					3.85*	3.46*
1368905_at *Ces2c* (includes others) carboxylesterase 2C			1.68**	6***	18.53***		2.15	3.29*		10.86***	14.86***		1.96	3.37*		9.29***	7.19***
1387328_at *Cyp2c9* cytochrome P450 family 2 subfamily C member 9							1.66*	1.73*	1.73	3.66**	3.94**			2.56		3.6	3.54
1387022_at *Aldh1a1* aldehyde dehydrogenase 1 family member A1	1.2		1.47**	2.49***	4.04***		1.96	3.96**		2.95***	3.72***					2.42*	2.65*
1368718_at *Aldh1a7* aldehyde dehydrogenase family 1, subfamily A7				9.7	15.45			1.29		2.91*	6.16**					3.01*	4.76*
1370067_at *Me1* malic enzyme 1				1.7**	3.12***			1.78*		2.77*	4.08**			2.33*		3.83*	4.67*
1370870_at *Me1* malic enzyme 1				2.04**	3.83***			1.8*		2.7*	3.94*			2.12*		3.12	3.83*
																	
*Conjugating Enzymes*																	
1369850_at *Ugt2a1*			1.24*	1.32*	2.06***		1.46	1.63*		1.72*	2.14*		1.5	1.98*		2.33*	2.3*
UDP glucuronosyltransferase family 2 member A1 complex locus																	
1370698_at *Ugt2b17*			1.7**	2.38***	2.73***		1.78*	1.74**		2.05***	2***		1.93*	2.05*		2.15*	2.34*
UDP glucuronosyltransferase family 2 member B17																	
1,387,955_at *Ugt2b17*					1.35*		1.67*	1.64*			1.33		1.81*	2.06*			1.63*
UDP glucuronosyltransferase 2 family, polypeptide B15																	
1,381,852_at *Ugt2b11*		-1.2		1.44**	3.26***			1.56						2.47*			
UDP glucuronosyltransferase family 2 member B11																	
1,368,397_at *Ugt2b7*			1.07	1.13**	1.2***		1.27*	1.25		1.28*	1.39*		1.35*	1.41*		1.31*	1.29*
UDP glucuronosyltransferase family 2 member B7																	
1,387,825_at *Ugt2b28*										3.57*	4.01*						6.15
UDP glucuronosyltransferase family 2 member B28																	
1,387,314_at *Sult1b1* sulfotransferase family 1B member 1	1.11			1.11	1.29**					3.74*	5.17**					2.83	4.22*
																	
*NrF2 antioxidant*																	
1,367,843_at *Akr7a2* aldo-keto reductase family 7 member A2			1.11	1.21*	1.39***			1.44*		1.44*	1.59**					1.36	1.6*
1,378,392_at *Dnaja3*				1.19*	1.25*		1.22	1.28*						1.54			
DnaJ heat shock protein family (Hsp40) member A3																	
1,387,669_a_at *Ephx1* epoxide hydrolase 1			1.26*	1.65***	2.13***		1.78*	2.28**		2.4*	2.94*			2.77*		3.46*	3.35*
1,368,180_s_at *Gsta1* glutathione S-transferase alpha 1			1.26	1.56**	2.11***		1.8	2.27*		2.05*	2.45*			3.38*		2.99*	3.61*
1,371,089_at *Gsta3* glutathione S-transferase alpha 3	1.39		1.67*	3.98***	13.87***		1.46	1.76*			1.9*			1.77*			1.91*
1,386,985_at *Gstm5* glutathione S-transferase mu 5			1.13	1.44***	1.86***			1.7*		1.46*	1.56*			2.44*		1.83*	1.92*
1,368,409_at *Gstt2/Gstt2B* glutathione S-transferase theta 2 (gene/pseudogene)					1.11		1.31	1.45*						1.74*			
1,367,613_at *Prdx1* peroxiredoxin 1				1.08*	1.27***			1.26**		1.19	1.21*			1.21*			
1,387,599_a_at *Nqo1*			1.39*	2.64***	6.62***								3.16	7.19*		2.6*	2.93*
NAD(P)H quinone dehydrogenase 1																	
1,374,070_at *Gpx2* glutathione peroxidase 2					1.65*			1.84		4.32*	6.38*						4.92
1,368,121_at *Akr7a3* aldo-keto reductase family 7 member A3				1.7*	3.21***			1.93			3					4.9*	6.92*
1,367,982_at *Alas1*				2.73**	4.04***		1.78*	2.88**		2.38*	2.68*		1.76	2.85*		2.75*	2.06*
5′-aminolevulinate synthase 1																	
																	
*Membrane Function*																	
1,369,455_at *Abcg5*				-1.89*	-3.99***					-3.45*	-6.29**						-3.05
ATP binding cassette subfamily G member 5																	
1,369,440_at *Abcg8*				-1.95*	-4.66***					-5.15**	-7.72***						-3.5*
ATP binding cassette subfamily G member 8																	
1,369,698_at *Abcc3*		1.35	2.09***	9.62***	23.41***		2.36*	2.83**		2.99*	3.42*		2.32	3.76*		3.4**	3.37**
ATP binding cassette subfamily C member 3																	
1,370,464_at *Abcb1*				1.87**	3.45***			1.86		2.1**	2.2**		2.28	3.1*		1.99	
ATP binding cassette subfamily B member 1																	
1,389,391_at *Slc35e3* solute carrier family 35 member E3				1.25	2.03***			1.38						1.6*			1.55
1,372,479_at *Slc4a4* solute carrier family 4 member 4						-1.43								1.77*			
1,368,191_a_at *Slc22a1* solute carrier family 22 member 1			1.12	1.27**	1.49***		1.53*	1.68*		1.38	1.44			1.56		2.13*	2.01*
1,368,600_at *Slc26a1* solute carrier family 26 member 1								1.6*									
1,395,325_s_at *Mmgt1* membrane magnesium transporter 1			1.26*	1.95***	2.54***		1.74*	2.28**		1.96*	2.22**		1.91	2.37*		1.91*	1.9*
1,376,168_at *Mmgt1* membrane magnesium transporter 1			1.2	1.81***	2.43***		1.66*	2.09**		2**	2.23**		1.89	2.13*		1.89*	1.82
1377854_at *Tmem62* transmembrane protein 62					1.21				1.29		1.34			1.61*		1.3	1.38*
1370807_at *Vmp1* vacuole membrane protein 1														1.54*			1.42*
1368977_a_at *Timm10b* translocase of inner mitochondrial membrane 10B				1.35**	1.81***			1.34		1.37	1.52*			1.51*			1.92
1387013_at *Cltrn* collectrin, amino acid transport regulator										11.24***	19.39***					10.7*	14.91*
																	
*Lipid/Protein Disease*																	
1371572_at *App* amyloid beta precursor protein			1.32*	2.43***	3.05***					2.64***	3.37***					3.01*	3.11*
1369727_at *Apoa2* apolipoprotein A2									1.78	3.68**	5.14**					3.15*	5.5*
																	
*Other*																	
1383585_s_at *Snx10* sorting nexin 10				3.53**	6.35***			1.39		3.09*	5.06*			2.29*		4.06	8.08*
1370371_a_at *Ceacam4* CEA cell adhesion molecule 4								-1.45								6.33*	25.18***
1370349_a_at *LOC100360095* (includes others) urinary protein 2																	45.56*
1370828_at *Zdhhc2* zinc finger DHHC-type containing 2			1.3	2.41***	7.17***					3.69***	6.17***					2.8*	4.68***
1377662_at *Pir* pirin				1.74**	3.44***		1.43	1.94*		1.83	2.36*			2.41*		2.02	2.77*
																	
*Cancer-Related, Cancer Driver Genes, & Mitochondria function*																	
1,369,957_at *Rgs5* regulator of G protein signaling 5				1.78**	1.75**												
1,383,288_at *Mdm2* MDM2 proto-oncogene				1.55**	2.32***					1.55*	1.66*			1.2			
1,383,420_at *Afg1l* AFG1 like ATPase			1.2	1.3*	1.3*												
1,387,109_at *Por* cytochrome p450 oxidoreductase				2.24*	3.85***		1.48	1.76*		1.76*	1.92*			2.49*		2.1*	2.52*
1,387,599_a_at *Nqo1* NAD(P)H quinone dehydrogenase 1			1.39*	2.64***	6.62***								3.16	7.19*		2.6*	2.93*
1,367,613_at *Prdx1* peroxiredoxin 1				1.08*	1.27***			1.26**		1.19	1.21*			1.21*			
1,398,310_at *Akr1d1* aldo-keto reductase family 1 member D1				1.2*	1.38***												
1,368,121_at *Akr7a3* aldo-keto reductase family 7 member A3				1.7*	3.21***			1.93			3					4.9*	6.92*
1,368,143_at *Anxa7* annexin A7				1.64**	3***					1.77*	2.31**					1.9*	2.79*
1,387,376_at *Aox1* aldehyde oxidase 1				1.32**	2.21***			1.73		1.45	1.66*					1.47	1.56
1,370,964_at *Ass1* argininosuccinate synthase 1				-1.37**	-1.63***		-1.84			-1.88*	-1.94*						
1,368,741_at *C9* complement C9				-1.16*	-1.2*												
1,367,733_at *Ca2* carbonic anhydrase 2				1.8**	3.46***					1.63*	1.89**						
1,382,603_at *Cd274* CD274 molecule				-1.69*	-2.16**												
1,371,810_at *Copg1* coatomer protein complex subunit gamma 1																	
1,384,334_at *Cps1* carbamoyl-phosphate synthase 1				-1.15*	-1.23**					-1.2	-1.21*						
1,387,083_at *Ctf1* cardiotrophin 1				1.27*	1.67***												
1,367,651_at *Ctsd* cathepsin D				1.19*	1.43***											1.58*	1.55*
1,386,904_a_at *Cyb5a* cytochrome b5 type A			1.13**	1.27***	1.45***		1.15	1.23*		1.21	1.27			1.33		1.42*	1.32
1,370,475_at *Cyp2b13/Cyp2b9* cytochrome P450, family 2, subfamily b, polypeptide 9				-1.1*	-1.29***												
1,368,607_at *Cyp4a22* cytochrome P450 family 4 subfamily A member 22				3.32**	4.36***					2.82*	2.57					3.85*	3.46*
1,371,622_at *Dph1* diphthamide biosynthesis 1				1.2*	1.17												
1,370,511_at *Fgb* fibrinogen beta chain				-1.1*	-1.24***									1.15			
1,383,288_at *Mdm2* MDM2 proto-oncogene				1.55**	2.32***					1.55*	1.66*			1.2			
1,370,831_at *Mgll* monoglyceride lipase				1.34*	2.26***			1.83									
1,378,506_at *Pik3c2a* phosphatidylinositol-4-phosphate 3-kinase catalytic subunit type 2 alpha																	
1,398,814_at *Rab11a* RAB11A, member RAS oncogene family				1.1*	1.27***					1.2*	1.31*						1.39
1,387,185_at *Apbb3* amyloid beta precursor protein binding family B member 3					-1.21						-1.38*						
1,398,375_at *Mta3* metastasis associated 1 family member 3			1.21	1.4**	1.43**		1.25	1.3			1.35*						
1,371,643_at *Ccnd1* cyclin D1					-1.45						-2.13*			-2.14*			-2.7*
1,381,748_at *Raph1* Ras association (RalGDS/AF-6) and pleckstrin homology domains 1				1.55*	1.78**					1.52	1.58*			1.96		1.53	
1,383,709_at *Rab8b* RAB8B, member RAS oncogene family				1.15	1.29**						1.29*						
1,372,017_at *Diablo/LOC100360940* diablo, IAP-binding mitochondrial protein				1.19*	1.35**				1.22	1.24	1.46*						
1,377,872_at *Mals1* mitochondrial assembly of ribosomal large subunit 1				1.25**	1.53***			1.21		1.41*	1.51*			1.36			1.66
1,373,074_at *Timm21* translocase of inner mitochondrial membrane 21				1.22*	1.36**					1.41	1.63*			1.3			
1,383,171_at *Tfb2m* transcription factor B2, mitochondrial				1.17*	1.28**					1.33*	1.32*						
1,372,927_at *Mrpl50* mitochondrial ribosomal protein L50			1.11	1.3**	1.37***				1.42	1.45*	1.54*						
1,368,977_a_at *Timm10b* translocase of inner mitochondrial membrane 10B				1.35**	1.81***			1.34		1.37	1.52*			1.51*			1.92
1,389,518_at *Tomm5* translocase of outer mitochondrial membrane 5				1.25*	1.4**												
1,369,023_at *Mipep* mitochondrial intermediate peptidase			1.13	1.24*	1.55***			1.31		1.28	1.42*						
1,372,105_at *Micu2* mitochondrial calcium uptake 2				1.21	1.26*					1.25	1.32*						
1,372,456_at *Mrps31* mitochondrial ribosomal protein S31				1.12	1.21*					1.22	1.33*						
1,373,982_at *Tefm* transcription elongation factor, mitochondrial					1.23*			1.28*	1.25	1.33*	1.31						
*Cancer Driver Genes*																	
1,370,563_at *Akr1c14* aldo-keto reductase family 1, member C14	1.16		1.23*	1.54***	1.86***			1.3*		1.31	1.44*			1.65		1.64*	1.63*
1,387,077_at *Arpp19* cAMP regulated phosphoprotein 19				1.42**	2.09***					1.49*	1.72*			1.59*			1.95*
1,377,855_at *Cep83* centrosomal protein 83				1.6**	2.06***			1.48		1.93*	2.21**		1.3	1.57*		1.56	
1,372,510_at *Srxn1* sulfiredoxin 1				1.57*	2.56***									1.91			2.6
1,371,113_a_at *Tfrc* transferrin receptor		1.66*		2.09**	6.02***												
1,384,169_a_at *Vav2* vav guanine nucleotide exchange factor 2				2.08***	3.02***					2.25*	2.3*			1.45		2.1*	2.06*
1,379,283_at *Braf* B-Raf proto-oncogene, serine/threonine kinase				1.19	1.19			1.24*									
1,382,264_at *Crebbp* CREB binding protein					-1.25*			-1.2*									
1,372,774_at *Entpd5* ectonucleoside triphosphate diphosphohydrolase 5 (inactive)				1.19	1.52***		1.28	1.71*						2.08*		1.85*	1.82
1,369,150_at *Pdk4* pyruvate dehydrogenase kinase 4								1.44*									
1,383,242_a_at *Cebpa* CCAAT enhancer binding protein alpha				-1.39	-1.6*						-1.79						
1,369,954_at *Idh1* isocitrate dehydrogenase (NADP (+)) 1					1.2*			1.23		1.14	1.23*						
1,370,957_at *Il6st* interleukin 6 signal transducer					-1.41												
1,376,082_at *Mecom* MDS1 and EVI1 complex locus																	-1.41*

Fold change values are present if they are significant for FDR <0.2. Asterisks indicate further levels of statistical significance (*FDR<0.05, **FDR< 0.001, ***FDR<10 < ^-10^). Annotations based on Ingenuity Pathway Analysis.

Of note was the upregulation of the Nrf2 antioxidant pathways, a pathway that is turned on as a defense when oxidative damage. This was particularly noted in the adult 5-day treatment group (Group One) including upregulation of *Nqo1* (NAD(P)H quinone dehydrogenase 1), *Akr7a3* (aldo-keto reductase family seven member A3), and *Ephx1* (epoxide hydrolase 1) (Table 3).

Membrane protein transcripts were upregulated including the transporters *Abcc3*, *Abcb1d*, solute carriers, and other ion transporters. *Abcg5* and *Abcg8* were significantly downregulated at PND 22 and in adult animals but not in PND 4 animals.

The transcriptomic signals for cancer were upregulated particularly in Group one (the 5-day adult exposure group) and included upregulation of protooncogene and oncogene transcripts (Table 2). The hepatocellular disease markers indicated activation of Ras signaling transcripts (e.g., *Rgs5*, *Rab11a*, and *Raph1*). These Ras disease pathway transcripts were not upregulated in the PND 4 or PND 22 groups after PBDE-47 or PBDE mixture exposures. Other cancer transcripts for genes “characterized” as cancer driver genes (Bailey et al., 2018) were upregulated in PND 4, PND 22, and adult exposure groups. Mitochondria function transcripts were significantly upregulated particularly in the adult exposure group.

After PBDE-47 exposure there were significant transcriptional changes at PND 4 and in adult animals at the lowest exposure level (0.1–0.2 mmol/kg; FDR ≤0.05), changes that were more pronounced than after comparable PBDE mixture exposures. This suggests that PBDE-47 may be more toxic at lower exposure levels than the PBDE mixture which contained lower amounts of PBDE-47. PBDE mixture exposure at 27–31 mmol/kg and 89–103 mmol/kg also had fewer significantly changed liver transcripts than the PBDE-47 exposure groups at these exposure levels.

**FIGURE 2:**
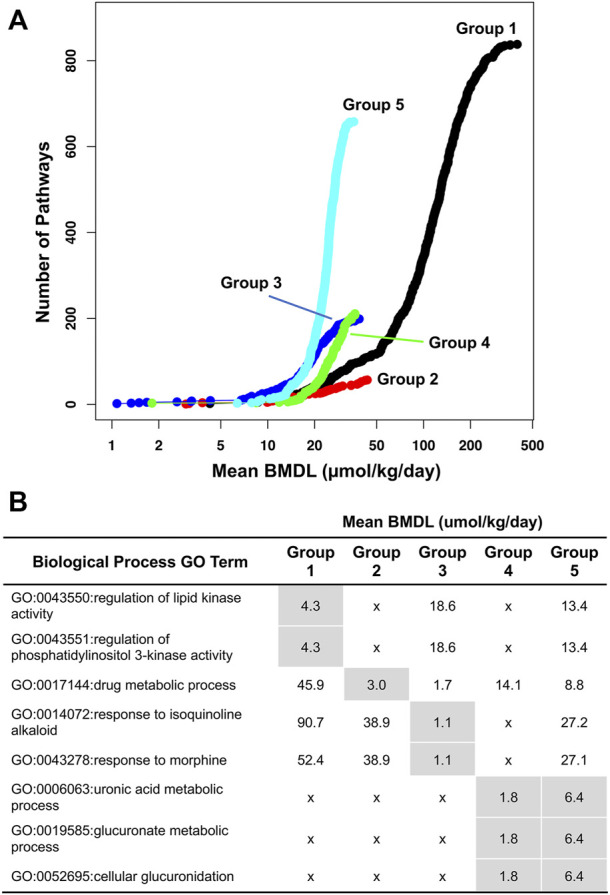
Toxicogenomic analysis of the five experimental datasets examined in this study. **(A)** Visualization of biological process gene ontology (GO) terms in concentration-response with the number of biological processes (*y*-axis) for a given level of the mean lower genomic benchmark dose (mean BMDL, *x*-axis). Group 1 (5 day PBDE-47 study) is shown in black, Group 2 (PBDE-47 at PND4) is shown in red, Group 3 (PBDE-47 at PND 22) is shown in blue, Group 4 (PBDE-mixture at PND4) is shown in green and Group 5 (PBDE-mixture at PND22) is shown in cyan. **(B)** The lowest mean BMDL for each group is shown in gray for each group for the most sensitive biological processes. An “x” indicates that a mean BMDL was not returned for a given biological process.

Male rat PBDE transcript data was used for Benchmark dose analysis (Figure 2). The BMDL (where there is estimated to be a 10% increase in toxicity over controls) was between ∼ 1 and 6 mmol/kg/day for PBDE exposures (for Groups 1–5). Lowest BMDL was based on GO categories with ≥3 genes (and ≥5% of the genes in the category being significant) that pass the selection criteria in BMDExpress 2.0 (*p*-value, ≥0.05 and |FC| > 1.5). Generally, these top GO categories were involved in various phases of metabolism including lipid metabolism.

## 4 Discussion

T4 levels are decreased in humans and animals after PBDE exposure (National Toxicology Program, 2015; Allen et al., 2016; Makey et al., 2016). Human exposure and body burden levels of PBDEs have been reviewed by the Agency for Toxic Substances and Disease Registry, Center for Disease Control (Agency for Toxic Substances and Disease Control, 2017), and they also report that PBDE exposure is associated with decreased blood levels of T4 in both humans and animals. These studies show that higher levels of PBDEs are needed in animals than in humans to cause decreases in T4 levels. Thus, humans may be more sensitive to this PBDE toxic effect than rodent models.

Transcriptomic changes in model systems can be used to discover biomarkers of disease and in risk assessment, and these data can provide an understanding of the relationship between molecular pathways and pathology of disease. This information can be used in risk assessment for linking pathologies and molecular pathways (Merrick, 2019; Szabo and Devlin, 2019; Sprenger et al., 2022).

Our analysis of previously published liver gene expression profiling data provides evidence that key characteristics of chemical carcinogens (Smith et al., 2016) were upregulated at PND 4, PND 22, and in adult male rats after exposure to the rodent chemical carcinogen, PBDE. The significant liver transcript changes at all three life cycle stages included upregulation of transcripts for metabolic, membrane function, and the *Nrf2* pathways, all characteristic of chemical carcinogens (Ma, 2013; Choi et al., 2021). These liver transcript changes after *in utero* exposure, neonatal exposure, or short-term adult exposures are biomarkers for upregulation of changes that can eventually lead to cancer.

The disruption of metabolic processes, as indicated by the PBDE-induced liver transcripts signals, is one of the key characteristics of chemical carcinogens (Smith et al., 2016; Guyton et al., 2018; La Merrill et al., 2020). This included upregulation of phase one and two liver metabolic enzymes (Table 3). Serum thyroxin (T4) levels were reduced in PBDE exposed groups, and this is attributed to PBDE-induced increase in levels of UGT transcripts, whose transcribed enzymes are used to catalyze the formation of thyroxine (T4) glucuronide which facilitates excretion of thyroid hormones in the bile (Richardson et al., 2008; Hoffman et al., 2017). Thyroid hormones were particularly low in the PND 4 rat because the development of thyroid production capacity is not yet fully developed at this life cycle stage (Dubois and Dussault, 1977; Choksi et al., 2003). If low levels of thyroid persist, this can promote development of hepatocellular cancer conditions (Frau et al., 2015; Lin et al., 2020).

PBDE metabolites can cause oxidative damage (Costa et al., 2014; Costa et al., 2015; National Toxicology Program, 2015). In this study, various *Nrf2* antioxidant pathway gene transcripts (Ma, 2013) were activated in response to PBDE treatment, particularly in the adult rat. The metabolic capabilities of younger animals may not yet be fully developed to complete all the metabolic steps necessary (MacLeod et al., 1972; Moscovitz and Aleksunes, 2013; O'Hara et al., 2015) to form the PBDE metabolites capable of causing oxidative damage (National Toxicology Program, 2015) and, thus, this may have contributed to the observed lower activation of *Nrf2* pathway transcripts in the younger animals.

Membrane transport protein transcripts were upregulated after PBDE exposure at all three life cycle stages. This included upregulation of xenobiotic efflux transporters and ion transporters. Upregulation of membrane transporters are characteristic of hepatocellular disease (Pedersen and Stock, 2013; Oosterwijk and Gillies, 2014; Prevarskaya et al., 2018), a disease that can become more severe with continued PBDE exposure (National Toxicology Program, 2015). For example, *Abcc3* transcript, an efflux transporter, was upregulated after PBDE exposure in the liver, and upregulation of this membrane transcript is characteristic of preneoplastic and neoplastic hepatocellular lesions in humans and rodents (Carrasco-Torres et al., 2016). Upregulation of membrane transporters is also characteristic of carcinogenesis processes in other organ systems (Donepudi et al., 2016).

The acidic nature of cancer cells is governed by ion transport (Swietach et al., 2014), and in this study many ion membrane transport gene transcripts were upregulated in all three life cycle stages after PBDE exposures. However, there was downregulation of the lipid efflux membrane transporter (*Abcg5/8*) (Patel et al., 2018) at PND 22 and in adult animals but not in PND 4 pups. Liver lipids levels in young animals are generally lower than in adult rodents (Smith and Abraham, 1970), and this may explain in part why lipid efflux pump transcripts were not changed in the PND 4 pups. Downregulation of ABCG5/8 can alter sterol excretion from cells (Patel et al., 2018).

Cancer and mitochondria function disease gene transcripts were upregulated in the adult rats (Table 3). This included Ras pathway transcripts *Rgs5*, *Rab11a*, *Rab8b*, and *Raph1* corresponding to human *RGS5* (Umeno et al., 2018; Abe et al., 2019; Shioga et al., 2020; Zhang et al., 2020), human *RAB11A* (Abd El Gwad et al., 2018; Cao et al., 2019), *Xenopus* and zebrafish *RAB8B* (Demir et al., 2013), and human *RAPH1* (Kurozumi et al., 2018). The RGS family is a group of multifunctional proteins that regulate cellular signaling events downstream of G-protein coupled receptors (Hurst and Hooks, 2009). *RGS5* expression is increased in multiple cancers (e.g., breast, ovarian, acute myeloid leukemia, and liver) and expression of *Rgs5* in rodents can also lead to other liver diseases (Bahrami et al., 2014). In addition, *Rgs5* expression in rodents promotes portal vein invasion and intrahepatic metastasis from hepatocellular carcinoma (Umeno et al., 2018).

The *Raph1* transcript was elevated after PBDE-47 exposure in the adult rat, a gene involved in cytoskeleton regulation (Kurozumi et al., 2018). High *RAPH1* expression has been correlated with aggressive breast cancer phenotypes and provides independent prognostic value in invasive breast cancer (Batistela et al., 2013). *Rab8b*, another member of the RAS family was also upregulated after PBDE exposure, and has been reported to be an essential evolutionary conserved component of Wnt/β-catenin signaling (Demir et al., 2013; Das et al., 2015). The elevated Ras pathway transcripts found in this study correlated with the finding of Ras mutations in PBDE-induced liver tumors in a 2-year cancer study (Dunnick et al., 2018b).

The overexpression of the *Mdm2* oncoprotein transcript after PBDE exposure seen in the adult rat in these studies, has been observed to frequently occur in hepatocellular carcinoma (HCC) (Wang et al., 2019). *MDM2* is an oncogene that is an inhibitor of the tumor suppressor, p53 (Ishizawa et al., 2018), and is an E3 ubiquitin ligase that directly binds to the N-terminal 1–42 aa of p53 to induce ubiquitin-mediated proteasomal degradation (Chen et al., 2019). *Mdm2* inhibits p53 translocation from the nucleus to the cytoplasm and enhances p53 degradation *via* ubiquitin-proteasome pathway (Soussi and Kroemer, 2018). About half of all cancers retain wild type p53, however the p53 pathway may be inactivated due to the overexpression of endogenous negative regulators, including the murine double minute 2 (*MDM2*), as occurred in PBDE treated rats in this study.

Generating enough energy is essential for cancer processes to proceed and critical to this is mitochondria function (Sokol et al., 2014; Porporato et al., 2018). In this study, mitochondria transcripts were upregulated in the adult animals. This included upregulation of genes encoding TIMM proteins, which have been characterized as diagnostic markers of poor prognosis for surviving cancer (Sotgia and Lisanti, 2017). The TIMM proteins, which are involved in the mitochondria protein-important machinery, are necessary for import of proteins needed for mitochondria function, but coded for in the nucleus (Pfanner and Meijer, 1997).

In short, the 5-day transcriptomics study of adult animals exposed to PBDEs offered comparable results to changes found after exposure in earlier life stages (PND4 and PND22). Furthermore, the analysis of this transcriptomic data identified alterations in toxic gene expression pathways predictive of longer-term toxic and carcinogenic effects using fewer animals and shorter exposure times than used in traditional toxicology studies (e.g., 13-week studies or 2-year bioassays). Thus, the 5-day transcriptomics study design helps to meet the objective to reduce, refine, and replace (3Rs) the use of animals in toxicology testing, an important national and international goal (National Toxicology Program, 2022).

More work is needed with other chemical carcinogens to compare transcriptomic changes at different life cycle stages, other target organs, and different model systems in order to determine the most appropriate conditions for predicting carcinogenic outcomes after short term exposures. Moreover, in this study we compared gene expression response with thyroid hormone levels and liver pathology. Future studies should likewise incorporate appropriate hematological and biochemical parameters, pathology, or other supporting data to complement and validate the transcriptomic approach.

## 5 Conclusion

This study presents liver gene expression profiles for a model chemical carcinogen (PBDE), that caused a liver carcinogenic response in rats and mice (National Toxicology Program, 2015), and also associated with toxicity in humans including lower thyroid hormone levels and developmental toxicity (Lam et al., 2017). Our results based on a combined analysis of previously published studies demonstrated that disease pathways are activated at all life cycle stages after PBDE exposure.

We show that for PBDEs a 5-day exposure period with transcriptomic endpoints could reduce the amount of time needed to identify toxic and carcinogenic potential compared to the traditional 13-week study, while using a fewer number of animals. More work is needed to understand whether the results presented here could similarly be used to reduce the number of animals needed to obtain toxic and cancer hazard information for other chemicals or classes of chemicals. If so, a similar transcriptomic assessment approach could be used to provide information on the underlying mechanisms.

Humans may be exposed to brominated chemicals early in life is a concern for adverse health effects (Vuong et al., 2018; Margolis et al., 2020; Varshavsky et al., 2020; Vuong et al., 2020), because early exposures can lead to disease later in life (Ferguson et al., 2017; Center for Disease Control, 2021). Other rodent model studies showed that short-term exposure to brominated chemical can lead to cancer later in life (Dunnick et al., 1997), and that the resultant tumors are characterized by alterations in the Ras pathway (Ton et al., 2004). These and other DTT studies suggest that further work is needed to characterize cancer hazards from exposure to mixtures of brominated chemicals (Peng et al., 2017; Dhungana et al., 2019; Han et al., 2020).

## Data Availability

Publicly available datasets were analyzed in this study. This data can be found here: The datasets analyzed for this study can be found on the National Library of Medicine’s GEO database web site (https://www.ncbi.nlm.nih.gov/geo/). The Affymetrix data for each dataset can be accessed from GEO using GSE153366 for Group One, GSE154914 for Group Two and Group Four, and GSE124431 for Group Three and Group Five.
